# Case Report: Development of medication-related osteonecrosis of the jaw in a patient on long-term infliximab therapy

**DOI:** 10.3389/froh.2024.1427060

**Published:** 2024-07-09

**Authors:** Derek Oryniak, Meagan Brown, Lillie Cholakis, Reda Elgazzar

**Affiliations:** Dr. Gerald Niznick College of Dentistry, University of Manitoba, Winnipeg, MB, Canada

**Keywords:** denosumab, MRONJ, Osteonecrosis of the Jaw, segmental resection of the jaw, Crohn's disease

## Abstract

Medication-Related Osteonecrosis of the Jaw (MRONJ) is a challenging and evolving aspect of Oral and Maxillofacial Surgery. In recent years, several medications apart from those traditionally associated with MRONJ such as bisphosphates (BPs) and Denosumab (DMB) have been implicated in bony necrosis of the jaw. This aim of this report is to demonstrate a significant case of bone necrosis following dental extractions on a patient being treated with infliximab therapy for Crohn's disease. Several cases in literature have reported MRONJ associated with infliximab but very few patients have developed as significant a form of the disease as seen in this report. Previous investigators have proposed pathophysiological pathways via which TNF-α inhibitors such as infliximab have a causative mechanism for MRONJ. When osteoclastic activity is restricted via these pathways, bone healing is impaired and MRONJ can occur. However, it remains a diagnostic challenge to differentiate between antiresorptive MRONJ and chronic osteomyelitis with bone necrosis in patients with acquired immunodeficiency. This case aims to illustrate why the antiresorptive effects of TNF-α inhibitors need to be considered as a possible primary driver of bone necrosis in such patients.

## Introduction

The definition of Medication-Related Osteonecrosis of the Jaw (MRONJ) has evolved in recent years and now encompasses other agents that can lead to the clinical findings of bony necrosis of the jaw following dental extractions. The most recent reiteration of the American Academy of Oral and Maxillofacial Surgeon's (AAOMS) Position Paper on MRONJ reflects this new research in its acknowledgment of the numerous medication families that have recently been implicated in the pathogenesis of MRONJ (1). A challenging diagnostic circumstance then arises as some of these same drugs, such as TNF-α inhibitors, primarily act as immunosuppressants, therefore also putting patients an increased post-operative infection risk. It is imperative clinicians understand the possible antiresorptive pathophysiology of these drugs to accurately diagnose and treat their patients.

Traditional antiresorptive medications such as bisphosphonates (BPs) and Denuosumab (DMB) have a well-established association with MRONJ (1). These drugs can cause necrosis of the jaw through bone remodeling inhibition and have direct effects on osteoclast formation, differentiation, and function. They are used in low doses for the treatment of osteoporosis and in higher doses for primary malignancy and bone metastasis to decrease Skeletal Related Events (SREs), including hypercalcemia of malignancy, reduction of bone pain and improvement of quality of life (2–4). Additionally, RANK ligand inhibitors and Romosozumab, used for the treatment of giant cell tumors and for fracture prevention respectively, also have demonstrated association with MRONJ (1).

Innate or acquired immune dysfunction places a patient at higher risk of MRONJ. Patients with medical co-morbidities such as diabetes, rheumatoid arthritis or immunocompromised states are at higher risk of developing MRONJ with or without exposure to antiresorptive agents (1, 5, 6). Numerous immune modulating drugs medications are known to increase a patients infection risk and are thought to have a synergistic effect with antiresorptive medications in the causation of MRONJ (1). However, TNF-α inhibitors, a specific class of immune modulating drug, demonstrate a pathway that directly implicates them with poor bony healing. Under normal function, TNF- α works to directly promote RANKL production by stromal cells and induces its secretion by T-Lymphocytes, B lymphocytes and endothelial cells to induce osteoclast formation. By blocking this pathway, a similar antiresorptive effect to those seen in bisphosphonates and Denosumab may been elicited by these drugs (7, 8).

In addition to drug and patient factors, there exists well-established local factors for the development of MRONJ. Predominately, these are related to dentoalveolar operations. A dental extraction is often implicated as the inciting event, however dental implants or other forms of dentoalveolar surgery have also been documented as a trigger (9, 10). Concomitant oral disease is also cited as a risk factor for the development of MRONJ, namely severe periodontal disease (10, 11). However, it must be noted that since severe periodontal disease is often the indication for dental extraction the relationship between the two factors and the development of MRONJ is challenging to establish. Genetic factors, inflammation or infection, and angiogenesis inhibition are also known to play a role in the development of MRONJ (1, 12, 13).

As noted in the 2022 update of the AAOMS position on MRONJ, multiple new classes of medications have now been implicated in the development of MRONJ (1). The mechanism by which these other classes of drugs may cause MRONJ is not explicitly stated, and may vary from the traditional form of MRONJ seen with antiresorptive medication. This case report attempts to differentiate between an infectious process exacerbated by an immunosuppressed state and the antiresorptive pathophysiology of MRONJ seen in a patient taking a TNF-α inhibitor. It also demonstrates how these drugs can be implicated in bone necrosis through there inhibition of RANKL production and thus osteoclast differentiation.

Ethical approval was granted by the Rady Faculty of Health Sciences (RFHS) Research Ethics Board (REB) to allow the creation of this case report after consent was obtained from the patient following established REB case report guidelines.

## Case report

In June 2022, a 78-year-old male was sent to the Oral and Maxillofacial Surgery clinic at the Health Science center in Winnipeg on referral from a community dentist due to right sided facial swelling of presumed odontogenic origin. The patient's medical history is significant for Crohn's Disease, Benign Prostate Hyperplasia, Hypertension, Dyslipidemia, and GERD. He had been taking infliximab via routine infusions for 10 years.

The patient had a pronounced, firm swelling at the right inferior border of his mandible. This correlated with a focal, erythematous and fluctuant swelling situated on the gingival mucosa buccal to tooth #46. Palpation of the buccal gingiva surrounding teeth #45 and #46 yielded purulent discharge from the marginal gingiva. A panoramic radiographic demonstrated widening of the periodontal ligament space around teeth #45, and #46. Tooth #46 was extracted without complication and an intraoral incision and drainage was performed with a sulcular incision.

Three weeks later, the patient presented to the OMFS clinic again with a persisting collection along the right inferior border of the mandible. The extraction socket of #46 had healed poorly with persistent granulation tissue. Purulent exudate was observed upon palpation of the buccal gingiva adjacent to tooth #45 from the gingival sulcus. The socket was curetted and tooth #45 was extracted. It was thought the patient was experiencing a standard post-extraction infection due to poor oral hygiene and his immunocompromised medical status. An aspirational biopsy was taken for cultures and sensitivities. Therapeutic intervention included placing the patient on an antibiotic regimen of Clindamycin 300 mg QID and 0.12% Chlorhexidine (Peridex) Oral Rinse.

**Figure 1:**
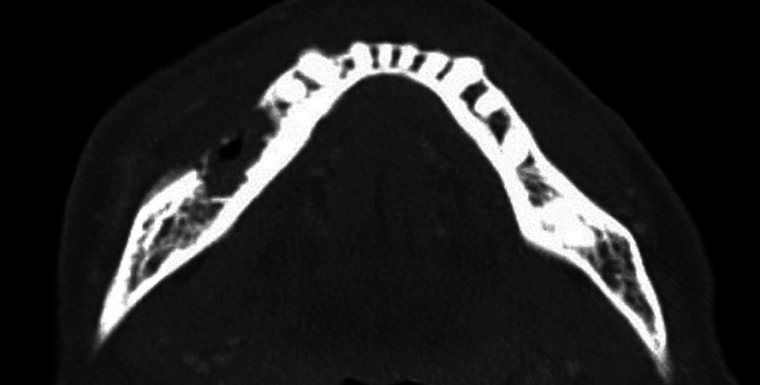
Panoramic radiograph. August 8th, 2022 (approximately 6 weeks post extraction). Minimal bone infill of extraction sockets #45, #46. No clear evidence of bone destruction in the region. Consistent with the lack of diagnostic ability of panoramic radiographs until 30%–40% of bone destruction has occurred.

The patient's progress was monitored with follow-up appointments scheduled at intervals of 2–3 weeks. In early August 2023, the extraction socket #45 had healed with epithelialization, however site #46, despite achieving peripheral epithelialization, continued to present with a small, unresolving, necrotic bony exposure. A panorex was taken which failed to reveal a significant bony defect (Figure 1). At this stage of treatment, differentiating between a prolonged post-extraction infection in an immunocompromised patient vs. MRONJ was challenging. However, the lack of purulence discharge associated with the necrotic bone led the investigators to treat this as antiresorptive process, rather than osteomyelitis. A tentative diagnosis of MRONJ was made by the investigators, related to the patient's TNF-α inhibitor. Infliximab treatment was suspended, and the patient was placed on pentoxifylline and tocopherol in addition to another round of oral antibiotic therapy and chlorhexidine mouth rinse.

**Figure 2:**
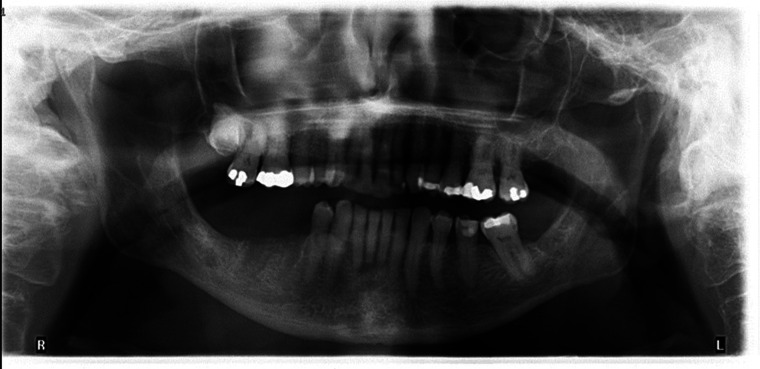
Axial slice CT facial bones and neck infused demonstrating areas of osteolysis with intramedullary gas formation. Appearance is concerning for osteomyelitis with cellulitis and areas of osteonecrosis originating from the site of extracted teeth in the lower right mandible.

In early November 2022, the patient presented to the clinic for a follow-up with new findings of right lower lip level B paresthesia (14), and persistent extraoral fistula with purulent drainage. A CT facial bones was obtained (Figure 2), showing areas of fragmented bone which contained intramedullary gas. Associated enhancing phlegmonous material was present within the mandibular foramen and propagated out of the mental foramen. There was an associated perimandibular and partly subperiosteal abscess with a sinus tract draining to an area of skin thickening in the right lateral submental soft tissues. In accordance with the AAOMS staging system, the patient's MRONJ received a classification of Stage 3 (1).

**Figure 3:**
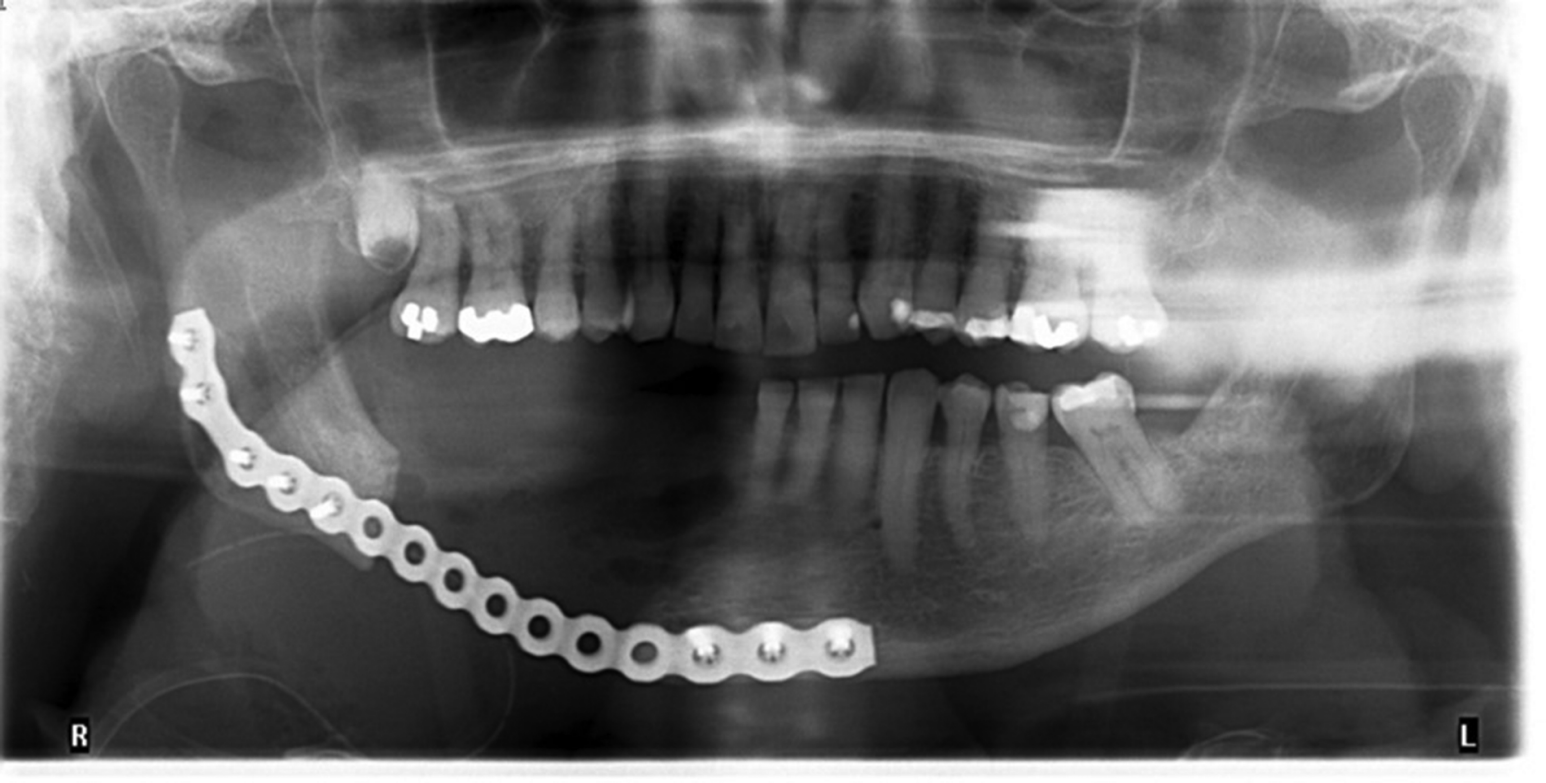
December 1st, 2022. Panoramic radiograph showing the extent of segmental mandibulectomy and 2.8 mm reconstruction plate in place. Good adaptation of place with 5 screws in proximal segment and 3 screws in distal segment.

In late Nov 2022, the patient underwent a right segmental mandibulectomy with removal of a four-centimeter segment of necrotic bone and fistulectomy, followed by reconstruction plate adaptation under general anesthesia. Unfortunately, at one week follow-up, the extraoral incision had dehisced at the site of the fistulectomy and there was a 1 cm intraoral dehiscence at the most posterior aspect of the incision. A repeat CT revealed developing cellulitis at the site with no defined collection. The patient was admitted for intravenous antibiotic therapy for 1 week after which the purulent drainage stopped and he was then discharged on a community intravenous antibiotic program in consultation with surgical infectious diseases. A post-operative panoramic radiograph demonstrated a good adaptation of the reconstruction plate (Figure 3).

Following a 6-week course of intravenous antibiotic treatment, the patient continued to have an extraoral fistula with significant purulent drainage. In consultation with surgical infectious diseases, it was decided to remove the fixation hardware. The patient was referred to the microvascular team for free flap reconstruction. However, due to the associated morbidity of free flap reconstruction the patient refused treatment and opted to receive a platysma flap for hardware coverage. Three months post-operatively the patient was doing well, with no recurrence of fistula or hardware exposure. Routine follow-up has confirmed no recurrence of infection or bone necrosis.

## Discussion

This case represents a challenging diagnostic exercise. Initially, the patient developed a minor infection which then resolved, then later presented with significant bone necrosis. Months after this initial sterile necrosis a significant bony defect was present which then became secondarily infected. The patient demonstrated poor wound healing post segmental mandibulectomy, eventually requiring a platysma flap to correct soft tissue dehiscence. Differentiating between a bone necrosis secondary to antiresorptive medication vs. a chronic osteomyelitis is difficult and the patients repeated healing difficulties must be separated from his initial disease process to understand what factor led to significant bony necrosis. Literature on the pathophysiology of MRONJ as it related to infliximab and other TNF-α inhibitors led the investigators to treat this as antiresorptive medication driven bone necrosis process related to the inhibition of osteoclast differentiation.

As reported in the most recent update of the AAOMS Position Paper on Medication-Related Osteonecrosis of the Jaw, several medications show a traditional antiresorptive mechanism association with MRONJ. These include bisphosphates (BPs), Denosumab (DMB), RANKL-inhibitors, and more recently Romosozumab (1). Notable in the updated position paper is that several other medications may also be a risk factor for MRONJ. These include tyrosine kinase inhibitors (TKIs), monoclonal antibodies such as bevacizumab, fusion proteins (aflibercept), mTOR inhibitors (everoliums), radiopharmaceuticals (radium 223), selective estrogen receptor modulators (ralxifene), and immunosuppressants (methotrexate and corticosteroids) (1). While these other drugs may be a risk factor for MRONJ, evidence is currently limited to isolated case reports and no established pathophysiology is outlined. Due to many of these patients' poly-pharmaceutical management and other risk factors, these various other pharmaceuticals are not formally listed as causative agents of MRONJ (1). However, other investigators have been more agreeable to directly implicate other drugs, namely TNF-α inhibitors, in cases of MRONJ (15–17).

The case discussed in this report represents a significant presentation of suspected MRONJ related to the long-term use of a TNF-α inhibitor for the treatment of Crohn's disease. TNF-α inhibitors have been reported to cause MRONJ following dental extractions, however few have demonstrated the extensive destruction seen in this report. In their 2020 review, Sacco and colleagues found 6 cases in the literature between 2006 and 2019 of patients who experienced MRONJ following exodontia or dental implant while receiving a TNF-α inhibitor. For three of the cases, the drug responsible was noted to be infliximab, consistent with the causative agent in this report (15). It should be noted, the majority of these cases represented mild cases MRONJ with staging not reported or stage 0, and all but one were treated with conservative management or simple debridement of necrotic bone to complete resolution (15).

In their retrospective report on the association of osteonecrosis of the jaw in patients being treated with TNF-α inhibitors, Brijs and colleagues reviewed 2,701 patients with inflammatory bowel disease (IBD) and cross-matched them with patients who met the criteria for MRONJ. Of these cases, 3 patients met the criteria for MRONJ and had no concomitant treatment with bisphosphonates (16). In the report, all 3 patients had been treated with infliximab for between 7 and 22 years. The cases represented stages 1, 2, and 3 MRONJ, and were treated via sequestrectomy, abscess drainage and sequestrectomy, and debridement and sequestrectomy respectively. Initial treatment was successful in all patients with one patient having a recurrence after 4 years (16).

The case discussed in this report represents an advanced form of MRONJ, meeting the criteria for stage 3 as per the AAOMS position paper due to the presence of an extraoral fistula and osteolysis extending to the inferior border of the mandible (1). In a review of the available literature, only a single case was described which required similar extensive intervention. Favia and colleagues discuss a patient who presented with a wide cutaneous necrotic area of her anterior mandible approximately 2 months following the extraction of 3 mandibular teeth. The patient underwent successful surgical treatment with wide bone resection and debridement of necrotic tissues (18).

In rheumatology research, significant attention has been given to the complication risk following surgery for patients taking TNF-α inhibitors. A 2007 study examining the topic found no significant association in the perioperative use of TNF-α inhibitors and infective comlications (19). Similar results were seen when examining patients with rheumatoid arthritis undergoing foot and ankle surgery, where no differences in healing or increased infection were seen in patients taking TNF-α inhibition agents (20). However, Neven and collogues did show a slight infection risk increase in patients undergoing orthopedic surgery when taking TNF-α inhibitors (21).

While disputed that TNF-α inhibitors significantly increase surgical site infection, this does not exclude an antiresorptive mechanism leading to bony destruction following dental extraction. Several investigators have noted pathophysiological mechanisms that implicate TNF-α inhibitor as a possible causative agent of MRONJ via an antiresorptive mechanism, similar to those seen with bisphosphonates. TNF-α promotes RANKL production by stromal cells and induces its secretion by T-Lymphocytes, B lymphocytes and endothelial cells to induce osteoclast formation indirectly (7, 8). TNF- α can also act directly on osteoclast precursors to potentiate RANKL-induced osteoclastogenesis in the absence of RANKL (22). This complex interplay between TNF- α, RANKL, and osteoclast formation and maturation indicate that disruption of these processes by TNF-α inhibition can have negative consequences on bone healing. As suggested by Brijs and colleagues, the inhibition of TNF-α would interfere with normal bone turnover, potentially leading to the development of MRONJ (16).

Differentiating between bone necrosis from antiresorptive medication and chronic osteomyelitis in an immunocompromised patient presented a significant challenge as described in this case report. The patient was consistently non-compliant with post-operative care after his initial dental extraction, and pre-existing periodontal disease led to early dehiscence of the site. It is difficult for the investigators to know the effect this poor management had on the infection site, and if they contributed to a concomitant skin infection. Clinical examination revealed extensive necrotic bone prior to the development of a secondary infection, indicating that a bone necrosis pathophysiology was likely the driver of the disease in this patient. The patient continually demonstrated poor healing following his segmental osteotomy, which necessitated further reconstruction. This poor healing must be separated from the initial bone necrosis however it cannot be ignored that several processes likely contributed to the patient's overall clinical picture, making a single definitive diagnosis elusive.

As previous investigators have demonstrated, dental extractions in patients undergoing treatment with TNF-α inhibitors such as infliximab can potentially lead to the development of MRONJ. This case report presents a severe case of MRONJ requiring extensive resection and provides a pathophysiologic mechanism to explain this necrosis in the setting of TNF-α inhibitor use. In conclusion, this case illustrated the challenging nature of defining MRONJ in medically compromised patients. This report documents an extensive bone necrosis which appears to have been primarily led by the patient's medication, rather than an infectious process.

## Data Availability

The raw data supporting the conclusions of this article will be made available by the authors, without undue reservation.
